# Metabolic characterization and metabolism-score of tumor to predict the prognosis in prostate cancer

**DOI:** 10.1038/s41598-021-01140-6

**Published:** 2021-11-18

**Authors:** Yanlong Zhang, Xuezhi Liang, Liyun Zhang, Dongwen Wang

**Affiliations:** 1 Department of Urology, First Hospital of Shanxi Medical University, 85 Jiefang Street, 030001 Taiyuan City, Shanxi Province China; 2 Department of Rheumatology, Shanxi Bethune Hospital, Shanxi Academy of Medical Sciences, 99 Longcheng Street, 030032 Taiyuan City, Shanxi Province China; 3 First Clinical Medical College, Shanxi Medical University, 56 Xinjian South Street, 030001 Taiyuan City, Shanxi Province China; 4 National Cancer Center/National Clinical Research Center for Cancer/Cancer Hospital and Shenzhen Hospital, Chinese Academy of Medical Sciences and Peking Union Medical College, No. 113 Baohe Road, Longgang District, 518116 Shenzhen China

**Keywords:** Cancer metabolism, Cancer models, Cancer therapy, Urological cancer, Bioinformatics, Gene expression analysis, High-throughput screening, Sequencing, Oncology, Urology

## Abstract

Tumor metabolism patterns have been reported to be associated with the prognosis of many cancers. However, the metabolic mechanisms underlying prostate cancer (PCa) remain unknown. This study aimed to explore the metabolic characteristics of PCa. First, we downloaded mRNA expression data and clinical information of PCa samples from multiple databases and quantified the metabolic pathway activity level using single-sample gene set enrichment analysis (ssGSEA). Through unsupervised clustering and principal component analyses, we explored metabolic characteristics and constructed a metabolic score for PCa. Then, we independently validated the prognostic value of our metabolic score and the nomogram based on the metabolic score in multiple databases. Next, we found the metabolic score to be closely related to the tumor microenvironment and DNA mutation using multi-omics data and ssGSEA. Finally, we found different features of drug sensitivity in PCa patients in the high/low metabolic score groups. In total, 1232 samples were analyzed in the present study. Overall, an improved understanding of tumor metabolism through the characterization of metabolic clusters and metabolic score may help clinicians predict prognosis and aid the development of more personalized anti-tumor therapeutic strategies for PCa.

## Introduction

Prostate cancer (PCa) is one of the most common male cancer types^1^. Although therapies, including laparoscopic radical prostatectomy and robot-assisted radical prostatectomy, for PCa have rapidly advanced in recent years, the recurrence rate is still high^2–4^. Simultaneously, almost all recurrent PCa cases become castration-resistant PCa (CRPC) after castration therapy^5^. Therefore, it is necessary to further explore the mechanisms underlying PCa to discover new treatments and seek biomarkers to predict prognosis and guide anti-tumor therapy in PCa.

The development and multiplication of cells are driven by the power of energy metabolism. Based on the characterization of the infinite proliferation of tumor cells, the emergence and growth of tumors is closely associated with the transformation of cell metabolic states. Recent studies have shown that the process of PCa emergence is related to tumor metabolism, including citric acid and choline metabolism^6^. Meanwhile, certain investigations have indicated that tumor metabolism associated with the androgen receptor (AR) leads to the occurrence and castration resistance of PCa^7,8^. It is possible to oppose this transformation toward PCa by inhibiting these metabolic pathways. Therefore, it is worthwhile exploring tumor heterogeneity and the mechanism underlying the metabolic perspective in PCa.

With the advent of high-throughput sequencing technology, tumor characterization of the whole genome can be performed. Compared with traditional metabonomic experiments, high-throughput sequencing technology can be used to analyze correlations between tumor metabolism and other biological behaviors, such as immune infiltration and tumor mutation burden (TMB)9 10. Therefore, highly comprehensive and accurate results can be obtained using high-throughput sequencing data and bioinformatics analysis.

Previous studies based on array and sequencing technology have reported that metabolic changes are the most obvious alterations in many types of cancer. In some cancers, high energy metabolism indicates a high capacity for proliferation and invasion as well as poor prognosis^11–13^. However, in specific tumors, including liver cancer, low metabolism indicates high tumor heterogeneity, which is associated with a poor prognosis^14^. The metabolic mechanisms underlying PCa development remain unknown.

Based on the information at hand, we investigated the tumor metabolic status using a large number of samples and multiple datasets with reasonable and complex bioinformatics methods. First, we collected PCa datasets from six databases, and 1,232 samples were included in our study. Then, unsupervised clustering analysis, principal component analysis (PCA), and univariate cox analysis to identify metabolic subsets and quantify tumor metabolic status based on the metabolic score were carried out. We further calculated the relationship between the metabolic score and tumor heterogeneity. We hypothesized that the metabolic score calculated based on metabolic gene expression from mRNA high-throughput sequencing or microarray data would better interpret PCa metabolic characteristics and be able to predict PCa prognoses while aiding anti-tumor therapy. The workflow of our research is shown in Fig. S1A.

## Materials and methods

### PCa datasets collected and preprocessing

We downloaded 1,232 tumor sample datasets from 1205 PCa patients (TCGA-PRAD, Deutsches Krebsforschungszentrum [DKFZ], and GSE54460 procured from the RNA-seq database; GSE70768, GSE116918, and Memorial Sloan-Kettering Cancer Center [MSKCC] procured from the Array Express database) from six publicly available databases. The samples in TCGA, DKFZ, GSE54460, GSE70768, and MSKCC were collected after radical prostatectomy, while the samples in GSE116918 were collected with primary radiotherapy (with or without ADT). Information on the clinical and data types of 1205 PCa patients is displayed in Table S1. The RNA sequencing data (FPKM: fragments per kilobase per million) of TCGA-PRAD datasets were collected from TCGA (https://portal.gdc.cancer.gov/). The RNA sequencing (FPKM) data of the GSE54460 datasets and the microarray datasets (GSE70768 and GSE116918) were collected from the Gene Expression Omnibus (GEO) (https://www.ncbi.nlm.nih.gov/gds/). The RNA sequencing (FPKM) data of the DKFZ datasets and the microarray datasets of the MSKCC datasets were downloaded from the cBioPortal for Cancer Genomics (http://www.cbioportal.org/). Details about each sample (including patient characteristics; treatments received; and methods of assay, preservation, and storage) can be obtained from the corresponding database websites. Then, we converted the expression profile (FPKM) of TCGA-PRAD, GSE54460, and DKFZ datasets to TPMs (transcripts per kilobase million) and processed TPM values with + 1 and log2 to ensure that these values were in accordance with the microarray values^15^. To eliminate batch effects between different datasets (GSE54460, GSE70768, GSE116918, MSKCC, and DKFZ cohorts), we used the "sva" R package and "ComBat" algorithm to produce the meta cohort (Fig. S1B and S1C)^16^.

### ssGSEA for metabolic pathways

Metabolic pathway gene sets collected by KEGG (Kyoto Encyclopedia of Genes and Genomes) were downloaded from the Molecular Signatures Database (MSigDB; https://www.gsea-msigdb.org/) (Table S2). The active levels of PCa samples in all the pathways were quantified by single-sample gene sets enrichment analysis (ssGSEA) using the "gsva" R package^17^. We employed unsupervised clustering and the "k-means (km)" method to identify the metabolic pattern of each PCa sample using the "ConsensuClusterPlus" R package and repeated it 1,000 times to ensure classification stability^18^.

### DEGs connected with metabolic subtypes

To further select genes associated with metabolic subtypes, we identified differentially expressed genes (DEGs) between these clusters through differential analysis using the limma R package (adjust *P*-value < 0.001).

### Construction of metabolic score and nomogram

First, we performed univariate cox analysis for each DEG to select those with prognostic potential. Next, we performed unsupervised cluster analysis based on the expression levels of the prognostic DEGs to classify patients in the meta cohort. Finally, PCA was performed based on the prognostic DEGs to describe the metabolic characteristics of the PCa samples. Principal components 1 and 2 were calculated as biomarkers to quantify the metabolic level of the PCa sample. The advantage of this approach is that it concentrates the score on the largest set of highly correlated (or unrelated) gene blocks in the set, while down-weighting the contribution of genes that are not tracked by other members in the set^19^.

The metabolic score was calculated as follows:

$${\text{Metabolic}}\;{\text{score }} = \, \Sigma \, \left( {PC1i \, + \, PC2i} \right)$$

where i is the expression of 216 prognostic DEGs.

Correlations among the clinical variates, metabolic score, and disease-free survival (DFS: biochemical recurrence-free survival) of PCa patients were analyzed using univariate cox analysis. The prognostic model and nomogram were constructed using multivariate Cox regression analysis. Kaplan–Meier (K–M) survival curves were used for prognostic analysis, and log-rank tests were performed to calculate P-values. To test the precision of the risk model and nomogram, time-dependent receiver operating characteristic (ROC) analysis was performed using the R package survivalROC 1.0.3. The area under the ROC curve (AUC) > 0.60 was considered to indicate that the prediction ability of the model was meaningful, and an AUC > 0.75 was considered to indicate outstanding predictive value.

### Functional enrichment analysis of the metabolic score

To investigate the potential molecular biological function of metabolic score genes, we performed gene set enrichment analysis (GSEA) in the meta cohort according to the high and low metabolic score groups divided by the medium metabolic score. We further explored the function of the metabolic score through gene ontology (GO) and Kyoto Encyclopedia of Genes and Genomes (KEGG) gene function enrichment analysis as well as prognostic DEGs (*P* < 0.05).

### Acquisition of DNA mutation data

We collected the DNA mutation and copy number variation (CNV) information of PCa patients in TCGA from TCGA data portal (https://www.cancer.gov/tcga/), and that for patients in the DKFZ cohort were downloaded from the cBioPortal for Cancer Genomics (http://www.cbioportal.org/). DNA mutation driver genes were selected for the high or low metabolic scores group using the "maftool" R package^20^. The top 20 driver genes with the highest mutation rates were investigated.

### Immune infiltration evaluation of PCa tumors

The related infiltration and activity levels of 29 immune cell gene sets collected from the Molecular Signatures Database (MSigDB; https://www.gsea-msigdb.org/) were calculated using the "gsva" R package (Table S2). The immune scores, stromal scores, and ESTIMATION scores calculated by the "ESTIMATE" R package were employed to evaluate immune cell and stromal cell abundance in PCa tumors^17^. To further investigate the relationship between stromal cells and the metabolic score, we calculated ssGSEA scores for epithelial-mesenchymal transition (EMT), extracellular matrix (ECM), and transforming growth factor-beta (TGF-β) using the corresponding gene sets downloaded from the Molecular Signatures Database (Table S2).

### Drug sensitivity analysis

To assess the connection between the metabolic score and drug sensitivity, we applied the "pROC" R package to predict drug IC_50_ by sample expression data. Bicalutamide, docetaxel, cisplatin, methotrexate, axitinib, doxorubicin, and gemcitabine were included in the analysis.

### Statistical analyses

All computational and statistical analyses were performed using the R software. The Wilcoxon test was used for the differential analysis of two groups. The Kruskal–Wallis test was used for more than two groups. Fisher's exact test was used to calculate the difference for contingency table variables, and the correlation coefficient of the two variables was calculated using Spearman correlation analysis. Statistical significance was defined as a two-tailed *P* value of < 0.05.

### Ethics approval and consent to participate

This study is based on published or public datasets and does not include new data that require ethical approval and consent.

### Consent for publication

Written informed consent for publication was obtained from all participants.

## Results

### Landscape of metabolic levels of tumors in PCa

First, we executed the ssGSEA algorithm to evaluate the activity levels of metabolic pathways in the PCa samples. Univariate cox and K-M survival curve analysis, based on 733 tumor samples with ssGSEA scores from the meta cohort (RNA-seq database: GSE54460 and DKFZ; array express database: GSE70768, GSE116918, and MSKCC), indicated that almost all metabolic pathways had a prognostic value in PCa (Table S3). Finally, we performed unsupervised clustering analysis to identify the metabolic patterns of PCa patients in the meta cohort.

**Figure 1:**
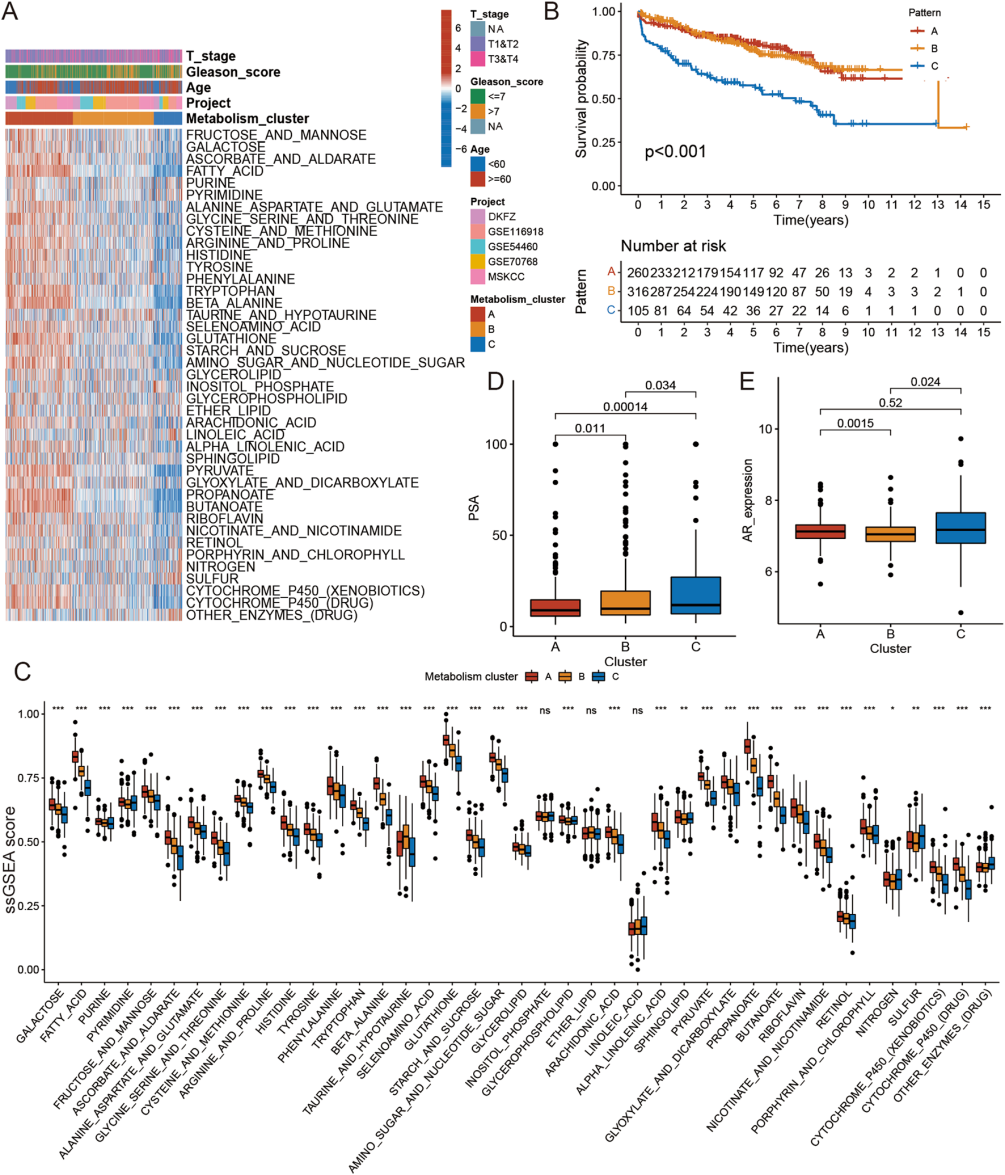
The landscape of active level of 41 metabolic pathways in PCa. (**A**) Unsupervised clustering of metabolic ssGSEA scores in five independent PCa cohorts. Rows represent tumor metabolic ssGSEA scores, and columns represent samples. (**B**) Kaplan–Meier curves for disease-free survival (DFS) of all PCa patients with metabolism clusters. The log-rank test showed an overall *p* < 0.001. (**C**) The metabolic ssGSEA scores in four metabolism clusters. The difference in PSA (**D**) and AR expression (**E**) among distinct metabolism clusters. The statistical difference of three metabolism clusters was compared through the Kruskal–Wallis test. *p < 0.05; **p < 0.01; ***p < 0.001.

We then confirmed three distinct metabolic subtypes with noticeable disease-free survival (DFS) differences (log-rank test, *P* < 0.001) (Fig. S2A–H; Fig. 1A and B). In addition, we generated a correlation coefficient heatmap to display the universal landscape of active metabolic pathways in PCa (Fig. S3). To further explore the possible biological differences that lead to prognostic differences among PCa patients, we compared the ssGSEA scores of the metabolic pathways of the PCa samples. Among the three metabolic subtypes, metabolic cluster C was associated with the worst prognosis. The main feature of cluster C was that almost all metabolic pathways had low activity levels (low-metabolic tumors). The main feature of cluster A was that almost all metabolic pathways of the samples had high activity levels (high-metabolic tumors), and cluster B contained metabolic pathways with medium activity levels (medium-metabolic tumors). We also analyzed androgen receptor (AR) and prostate-specific antigen (PSA) levels in each metabolic subtype. Metabolic cluster C displayed an obviously higher PSA level than clusters A and B. By contrast, cluster B had lower AR expression levels than clusters A and C. The Kruskal–Wallis test was used to identify significant differences between the metabolic pathways, ssGSEA scores, AR expression levels, and PSA levels in the three unique metabolic subtypes (Fig. 1C–E).

### Identification of metabolic gene subsets

**Figure 2:**
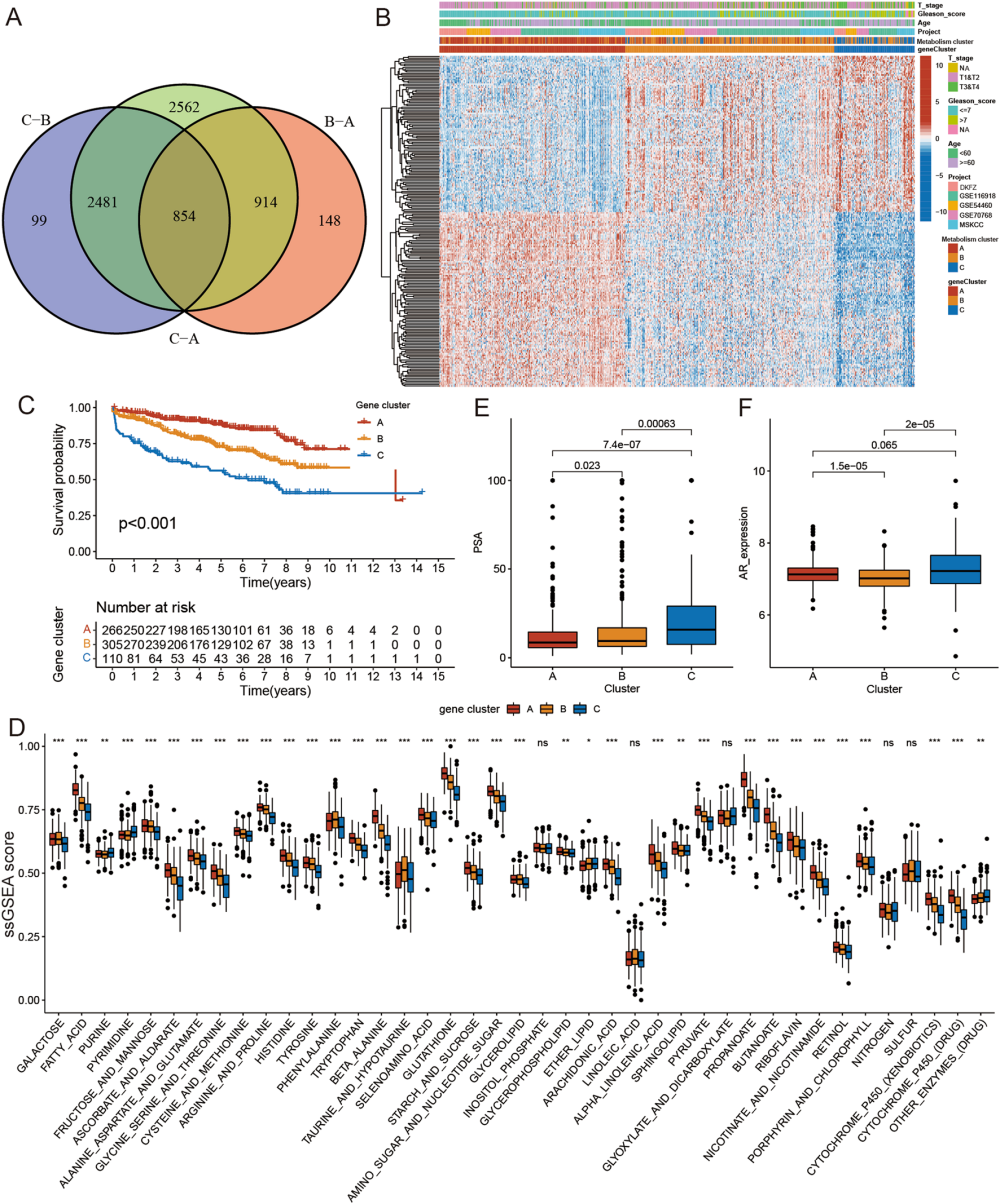
Identification of metabolic gene subtypes. (**A**) Venn diagram depicting 854 differentially expressed genes in three metabolism clusters. (**B**) Unsupervised clustering of DEGs among three metabolism clusters to classify patients into three groups: gene cluster **A–C**. (**C**) Kaplan–Meier curves for the three groups of patients. The log-rank test showed an overall *p* < 0.001. (**D**) The metabolic ssGSEA scores in three gene clusters. The difference in PSA (**E**) and AR expression (**F**) among distinct gene clusters. The statistical difference of three gene clusters was compared through the Kruskal–Wallis test. *p < 0.05; **p < 0.01; ***p < 0.001.

To reveal the potential biological properties of different metabolic subsets, we performed differential analyses to identify the transcriptome characteristic genes between the high, low, and medium metabolic tumors using the limma package of R software (Fig. 2A). Next, we selected DEGs with a prognostic value by univariate cox analysis, performed unsupervised clustering analysis again based on the expression levels of 216 prognostic DEGs (Tables S4 and S5), and divided the meta cohort into three gene clusters (gene clusters A, B, and C) (Fig. S4A–G). The heatmap describes the expression abundance of the 216 prognostic DEGs in three metabolic gene clusters (Fig. 2B). To explore the potential biological functions of these prognostic DEGs, GO and KEGG functional enrichment analyses were performed (Table S6). Functional enrichment analysis indicated that these prognostic DEGs play a role in cell metabolism and ion response.

Moreover, we compared the DFS of patients with PCa from different metabolic gene clusters using Kaplan–Meier survival analysis. The results suggested that patients in gene cluster A had a longer DFS and better prognoses than those in gene clusters B and C and accord to metabolism cluster A (log-rank test, *P* < 0.001) (Fig. 2C). We also discovered that gene cluster A had higher ssGSEA scores for most metabolic pathways. Therefore, we designate the samples corresponding to gene cluster A as high-metabolic tumors. Samples corresponding to gene clusters B and C were similarly designated as medium- and low-metabolic tumors. In addition, the three gene clusters displayed obvious differences in AR expression and PSA levels. For the PSA level, gene cluster C showed higher values than gene cluster B/C, and for AR expression, gene cluster B had lower values than gene cluster A/C. The Kruskal–Wallis test was applied to determine significant differences between the metabolic pathway ssGSEA score, AR expression levels, and PSA levels in the three gene clusters (Fig. 2D–F). Taken together, these results indicate that our classification method can extract metabolic characteristics and reflect the prognosis and tumor heterogeneity in PCa.

### Generation of the metabolic score

**Figure 3:**
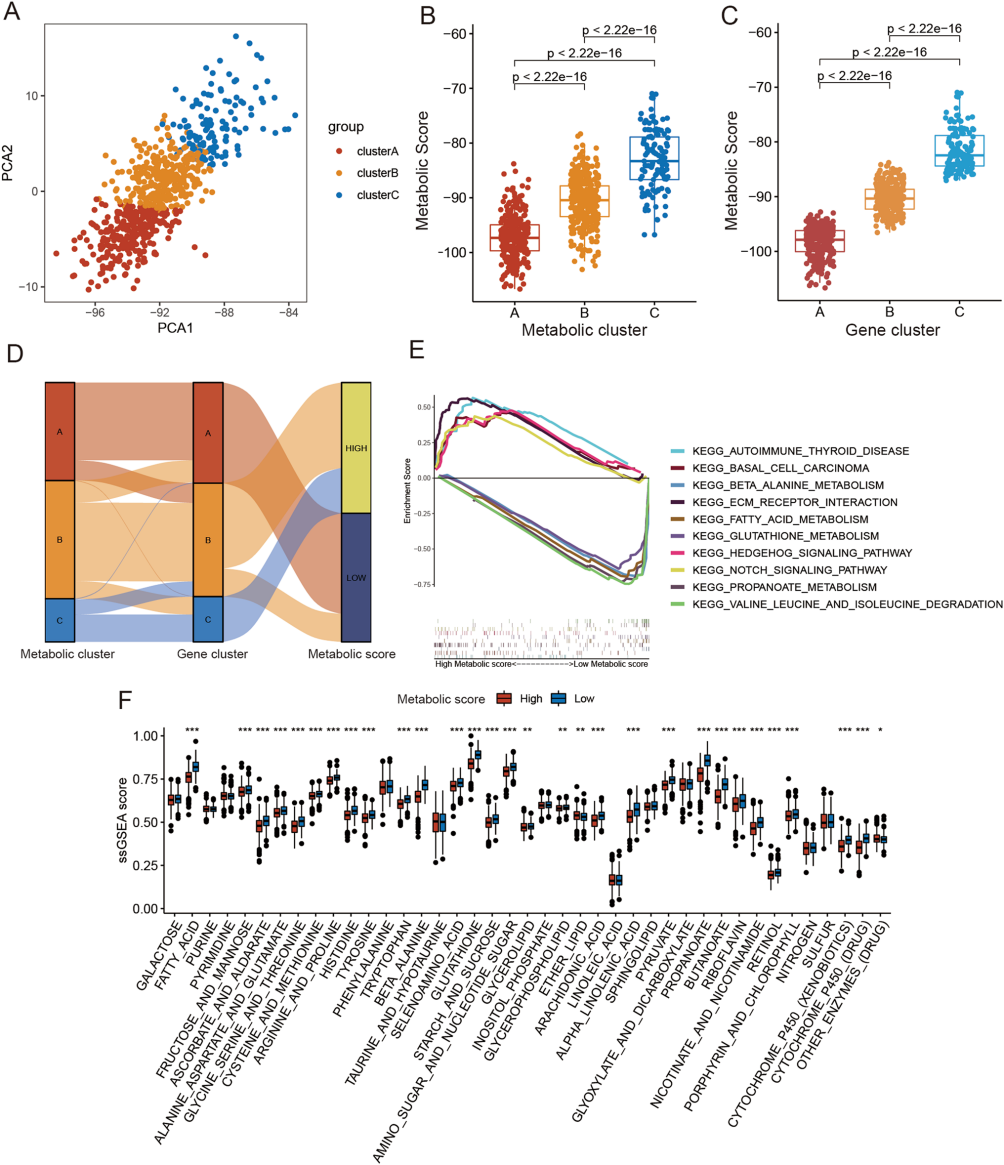
Construction of the metabolism-score. (**A**) Principal Component Analysis (PCA) based on metabolic gene signatures expression. (**B**) Boxplot of metabolic score for three metabolism clusters in the meta cohort. (**C**) Boxplot of metabolic score for three metabolic gene clusters in the meta cohort. (**D**) Alluvial diagram of metabolic gene cluster distribution in groups with different metabolism clusters, gene clusters, and metabolic score. (**E**) Enrichment plots showing autoimmune thyroid disease, base excision repair, ECM receptor interaction, intestinal immune network for IGA production, and notch signaling pathway in the low metabolism-score subgroup, and showing beta-alanine metabolism, butanoate metabolism, fatty acid metabolism, propanoate metabolism, and valine leucine and isoleucine degradation in the high metabolism-score subgroup. (**F**) The metabolic ssGSEA scores in high and low metabolic score groups. The statistical difference of two groups was compared through the Wilcoxon test. *p < 0.05; **p < 0.01; ***p < 0.001.

To obtain a quantitative biomarker of metabolic characterization in PCa patients, we produced the PCA algorithm to calculate the metabolic score, which was the value of the sum of PCA1 and PCA2 from the prognostic DEGs (Fig. 3A). The coefficient of the PCA is displayed in Table S7. We then divided the patients from the meta cohort into high and low metabolic score groups by cut-off values (medium metabolic score = − 92.30). The distribution of patients from the meta cohort in the three gene clusters is shown in Fig. 3B–D. Additionally, the GSEA results of the meta-analysis revealed that the Notch pathway was enriched in the low metabolic score group, whereas metabolic pathways were enriched in the high metabolic score group (Fig. 3E and Table S8). To further identify the metabolic feature of samples between high and low metabolic score groups, we compared the ssGSEA score of metabolic pathways and found that 30/41 pathways were significantly different (Fig. 3F). These results further suggest that the metabolic score can describe the metabolic characteristics of PCa samples.

We further compared the metabolic score between Africans and Caucasians in TCGA and MSKCC cohorts, for which there was no significant difference (Fig. S5A–B). Finally, we analyzed the relationship between the metabolic score and tumor metastasis. We found that patients with lymph node-positive tumors and distant metastasis had higher metabolic scores than those without (Fig. S5C–D). These findings indicate that the metabolic score can serve as a predictor for tumor metastasis.

### Validation of the prognostic value of the metabolic score and construction of nomogram

**Figure 4:**
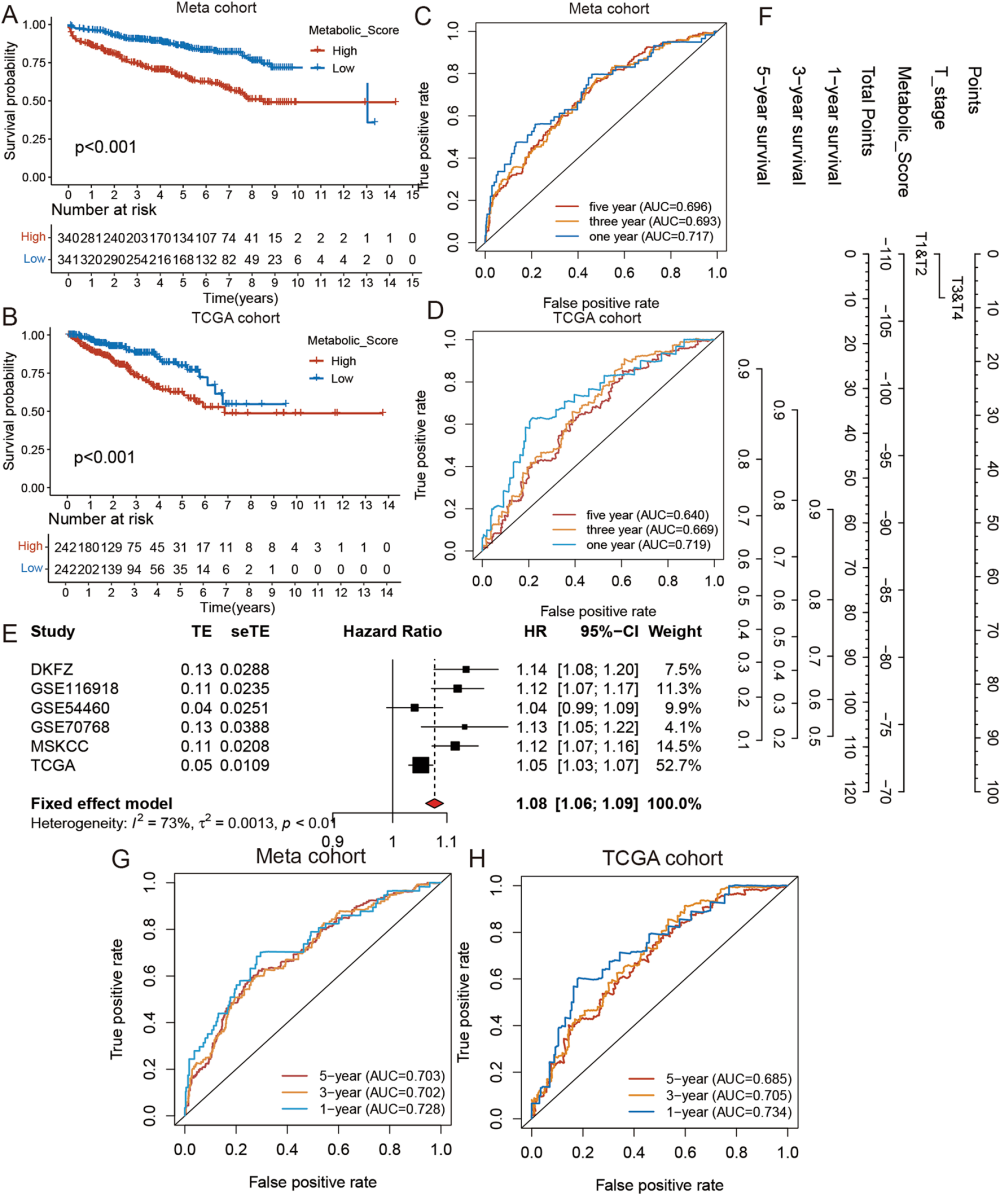
The prognostic value of metabolic score. (**A**) Kaplan–Meier curves for low and high metabolic score groups in meta cohort. Log-rank test, *p* < 0.001. (**B**) Kaplan–Meier curves for low and high metabolic score groups in TCGA cohort. Log-rank test, *p* < 0.001. (**C**) The ROC analysis of metabolic score in meta cohort. AUC = 0.717, 0.693, and 0.696 at 1, 3, and 5 year. (**D**) The ROC analysis of metabolic score in TCGA cohort. AUC = 0.719, 0.669, and 0.640 at 1, 3, and 5 year. (**E**) The meta-analysis of the HR of metabolic score in six cohort. (**F**) The nomogram based on metabolic score and clinical variates. (**G**) The ROC analysis of nomogram in meta (training) cohort. AUC = 0.728, 0.702, and 0.703 at 1, 3, and 5 year. (**H**) The ROC analysis of nomogram in TCGA (validation) cohort. AUC = 0.734, 0.705, and 0.685 at 1, 3, and 5 year.

Since we found a correlation between the metabolic score and prognosis, we divided the PCa patients into a meta-cohort, based on a median metabolic score (cut-off value = − 92.30), into high and low metabolic score groups and verified the prognostic value of the metabolic score by performing K-M survival curve analysis. The results of this analysis suggested that the patients in the low metabolic score group had a prolonged DFS time in the meta-cohort (log-rank test: *P* < 0.001) (Fig. 4A). We then divided the PCa patients into a TCGA cohort, based on a medicine metabolic score (cut-off value = − 77.13), into high and low metabolic score groups and further validated the prognostic value of metabolic score (log-rank test: *P* < 0.001) (Fig. 4B). ROC analysis also indicated that the metabolic score predicted the values in both the meta and TCGA PRAD cohorts and could predict DFS at 1, 3, and 5 years (Fig. 4C and D). We also validated the prognostic value of the metabolic score in each cohort included in the meta-cohort by K-M survival curve analysis. The results were consistent with the meta-analysis, and the low metabolic score group had a prolonged DFS in the GSE54460 (log-rank test: *P* = 0.038), GSE70768 (log-rank test: *P* = 0.224), GSE116918 (log-rank test: *P* < 0.001), DKFZ (log-rank test: *P *< 0.001), and MSKCC (log-rank test: *P* < 0.001) cohorts (Fig. S6A–E). A possible reason for the *P*-values of the GSE70768 cohorts being greater than 0.05 could be the small sample size. To further identify the predictive value of the metabolic score (continuous variable) in PCa, we performed univariate Cox analysis and meta-analysis to calculate the hazard ratio (HR) in six datasets (Fig. 4E). The results (HR = 1.08) also indicated that the metabolic score is a reliable prognostic marker.

This was further confirmed by performing a risk stratification analysis between the metabolic score (categorical variable; cut-off value = − 92.30) and clinical or traditional risk factors, including age, Gleason score, T stage, Cancer of the Prostate Risk Assessment (CAPRA) score, and National Comprehensive Cancer Network (NCCN) stage, in the meta cohorts (Fig. S7). The results confirmed that the metabolic score is an independent prognostic biomarker with clinical and traditional risk factors and can accurately predict DFS in patients with PCa. To improve the predictive value of the metabolic score, we selected clinical variables with independent prognostic values to obtain a nomogram through univariate and multivariate Cox analysis in the meta-cohort (Fig. 4F and Table S9). We used ROC analysis to assess the clinical significance of the metabolic score (continuous variable), clinical variate, and nomogram. Superior results were obtained for the metabolic score as a more accurate and reliable prognostic biomarker than clinical variates, while the nomogram had a better net benefit than clinical variate or metabolic score-only models (Fig. S8A–C). Furthermore, to independently validate the predictive value of the nomogram, we calculated the total points of each sample in the TCGA PRAD cohort and performed ROC and K-M survival curve analysis. Our findings on the efficacy of the nomogram demonstrated that it could predict DFS of PCa patients in both the meta and TCGA PRAD cohort (Fig. 4G–H, and S8D–S8E). Finally, we compared the area under the curve (AUC) of the ROC of our nomogram, CAPRA score, NCCN stage, TMB, and MSI and found that our nomogram had a better prognostic value than the other signatures (Fig. S8F–I).

### Relationship between the metabolic score and DNA mutations

**Figure 5:**
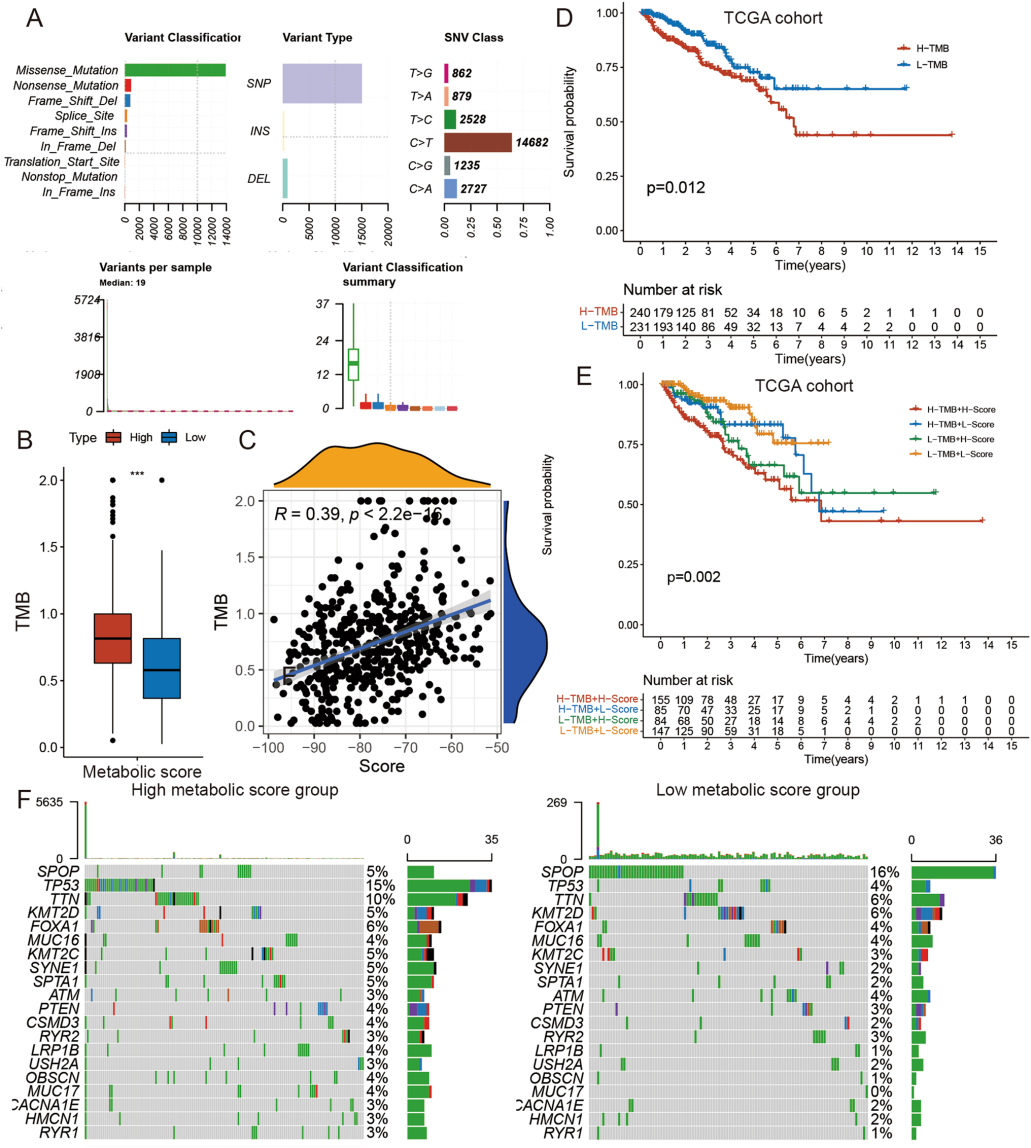
The Correlation between the metabolism-score and somatic variants in TCGA cohort. (**A**) The summary information of Somatic Variants in TCGA cohort. (**B**) TMB difference in the high and low metabolic score subgroups. Wilcoxon test, *p* < 0.001. (**C**) Scatterplots are depicting the positive correlation between metabolism-scores and mutation load. The Spearman correlation between metabolic scores and mutation load is shown (*p* < 0.001). (**D**) Kaplan–Meier curves for high and low TMB groups. Log-rank test, *p* = 0.012. (**E**) Kaplan–Meier curves for patients stratified by both TMB and metabolism-scores. Log-rank test, overall *p* = 0.002. (**F**) The oncoPrint was constructed using high metabolic scores on the left and low metabolic scores on the right. Individual patients are represented in each column.

The GSEA indicated that base excision repair (BER) was associated with the metabolic score. Simultaneously, many studies have shown that TMB is closely related to variations in tumor metabolism^21,22^. Through obvious clinical signs of TMB, we continued to investigate the relationship between TMB and metabolic scores to explain the genetic characteristics of each metabolic group in the TCGA cohort (Fig. 5A). First, the TMB was compared between the high and low metabolic score groups. We found that patients in the high metabolic score group displayed higher TMB than those in the high metabolic score group (Wilcoxon test: *P* < 0.001) (Fig. 5B). Through correlation analyses, we further found that the metabolic score was positively correlated with TMB (Spearman coefficient: R = 0.39, *P* < 0.001) (Fig. 5C). Thereafter, we divided the patients into high and low TMB groups based on the median TMB value. As shown in Fig. 5D, we found that patients with low TMB had better DFS than those with high TMB (log-rank test, *P* = 0.012). Next, to compare the predicted value of the prognosis of TMB and metabolic score simultaneously, we further divided the patients into four groups using medium TMB value and medium metabolic score. As expected, the group with low TMB and high metabolic score had the best prognosis, and the group with high TMB and low metabolic score had the worst prognosis (Fig. 5E, log-rank test) (*P* = 0.002).

Moreover, we evaluated the characteristics of DNA mutations in PCa driver genes between the low and high metabolic score groups. The top 20 highest mutation rate genes were further investigated (Fig. 5F). Analysis of the mutation data of TCGA cohort indicated that the DNA mutations of SPOP and TP53 were associated with the metabolic score groups. The SPOP mutation rate was higher in the low metabolic score group, while the TP53 mutation rate was lower in the low metabolic score group (Fisher's exact test; *P* < 0.05; Table 1). To further verify our results, we conducted the same analysis for the DKFZ cohort and observed that the results of the analysis of the DKFZ cohort were in accordance with those for the TCGA cohort (Fig. S9A–F). These results could provide novel ideas for exploring the mechanisms of tumor metabolism and gene mutations in PCa.

**Table 1:** Association of metabolic score with somatic variants.

Gene symbol	High metabolic score (%)	Low metabolic score (%)	*P*-value
SPOP	12 (5%)	36 (16%)	*P* < 0.0001
TP53	35 (15%)	9 (4%)	*P* < 0.0001

CNV is an upstream regulatory mechanism of mRNA transcription and leads to the genesis and development of cancer^23^. In a previous study, we established that CNV is related to the tumor metabolic status in PCa^22^. To further confirm the relationship between CNV and the metabolic score, we first compared the number of CNV amplifications and deletions of metabolic genes in the high and low metabolic score groups. The results indicated that the number of CNV amplifications and deletions was higher in the low metabolic score groups (Wilcoxon test [deletion]: *P* < 0.001; Wilcoxon test [amplification]: *P* < 0.001) (Fig. S10A). The correlation analysis suggests significant negative correlations between the number of CNV amplifications/deletions and the metabolic score (Spearman coefficient [amplification]: R = 0.33, *P* < 0.001; Spearman coefficient [deletion]: R = 0.51, *P* < 0.001) (Fig. S10B and C) and further suggests that CNV was the potential driving factor that led to the variation in tumor metabolic status and heterogeneity.

In previous research, MSI has been considered an important factor of genomic instability and has been demonstrated to be implicated in many genetic diseases. Our work involved downloading MSI information of the PCa samples predicted in the previous investigation and comparing it between the high and low metabolic score groups^24^. We found that the low metabolic score group had a higher MSI level than the high metabolic score group (Wilcoxon test: *P* < 0.001; Spearman coefficient: R = 0.22, *P* < 0.001) (Fig. S10D and E), indicating that MSI may also be a driving factor in the variation of tumor metabolic status and heterogeneity.

### Correlation between TME and metabolic score in PCa

**Figure 6:**
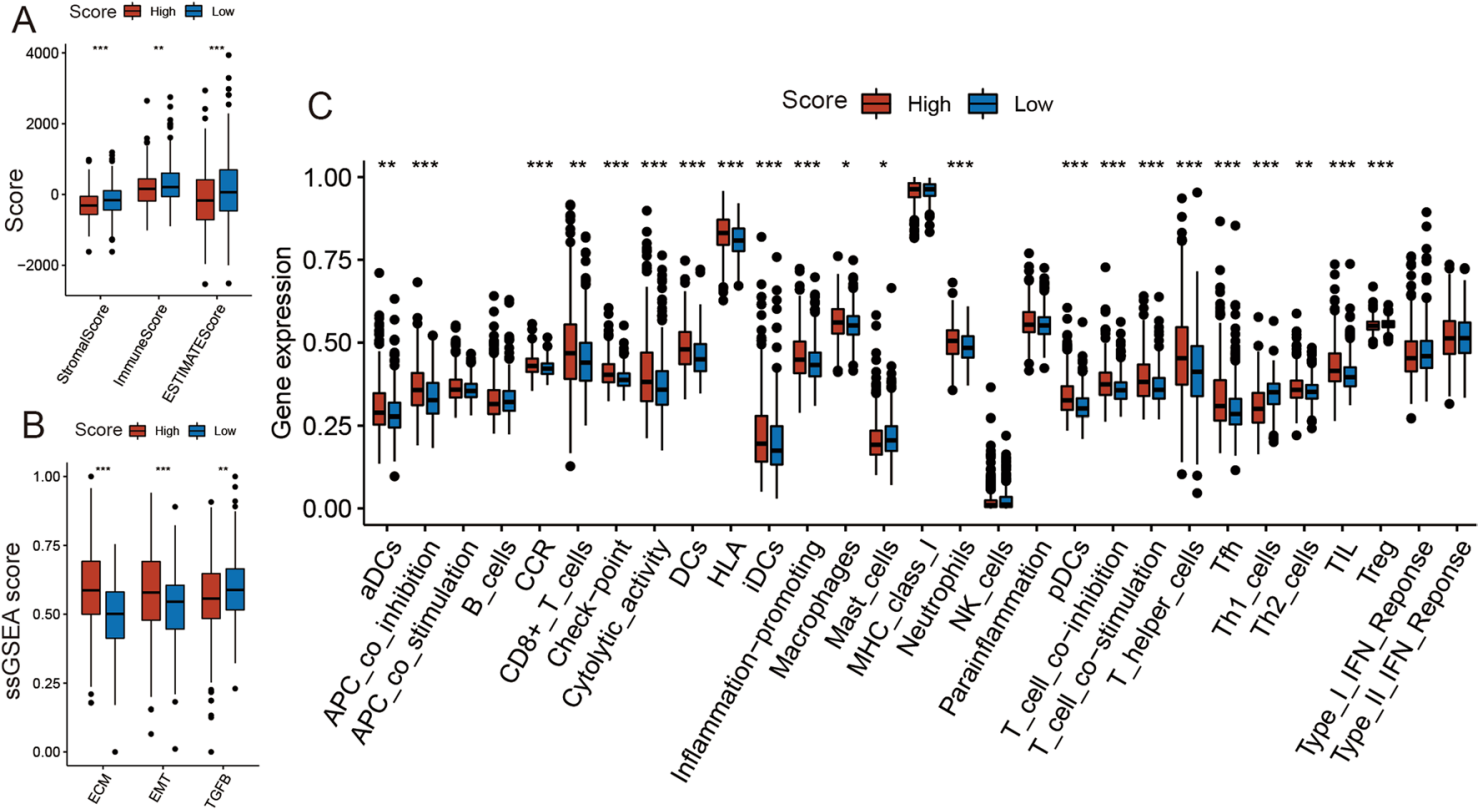
The Correlation between the metabolism-score and TME. (**A**) The stromal score, immune score, and ESTIMATE score differences in the high and low metabolism-score subgroups. (**B**) The ssGSEA score differences of ECM, EMT, TGF-β in the high and low metabolism-score subgroups. (**C**) The ssGSEA score differences of 29 immune pathways in the high and low metabolism-score subgroups. Wilcoxon test. *p < 0.05; **p < 0.01; ***p < 0.001.

TME includes tumor cells, immune cells, and other stromal cells and plays an important role in cancer development^25,26^. In a previous GSEA, we found that our metabolic score had a close relationship with cellular immune function. Therefore, we further analyzed the differences in TME between the high and low metabolic score groups. The result of the ESTIMATE analysis suggested that, regardless of stromal or immune cells, there was a significant difference between the two groups (Wilcoxon test [stromal score and ESTIMATE score]: *P* < 0.001; Wilcoxon test [immune score]: *P* < 0.01) (Fig. 6A). To further compare the stromal cells of the two groups, we used the ssGSEA score of EMT, ECM, and TGF-β pathways and found that the ssGSEA scores of ECM and EMT were higher in the high metabolic score group, while TGF-β was higher in the low metabolic score group (Wilcoxon test [ECM]: *P* < 0.001; Wilcoxon test [EMT]: *P* < 0.001; Wilcoxon test [TGF-β]: *P* < 0.01) (Fig. 6B). Then, to confirm which immune cells were different between the two groups, we compared the ssGSEA score of 29 immune pathways using the Wilcoxon test and found that ssGSEA scores of aDCs, APC_co_inhibition, checkpoint, CCR, CD8 + _T_cells, cytolytic activity, DCs, iDCs, HLA, inflammation-promoting, Macrophages, Neutrophils, pDCs, Tfh, T helper cells, Th2 cells, and TIL were significantly higher in the high metabolic score group, and the ssGSEA scores of Mast_cells, Th1_cells, and Treg were significantly lower in the high metabolic score group (Fig. 6C).

### Predictive value of metabolic scores in anti-tumor therapy

**Figure 7:**
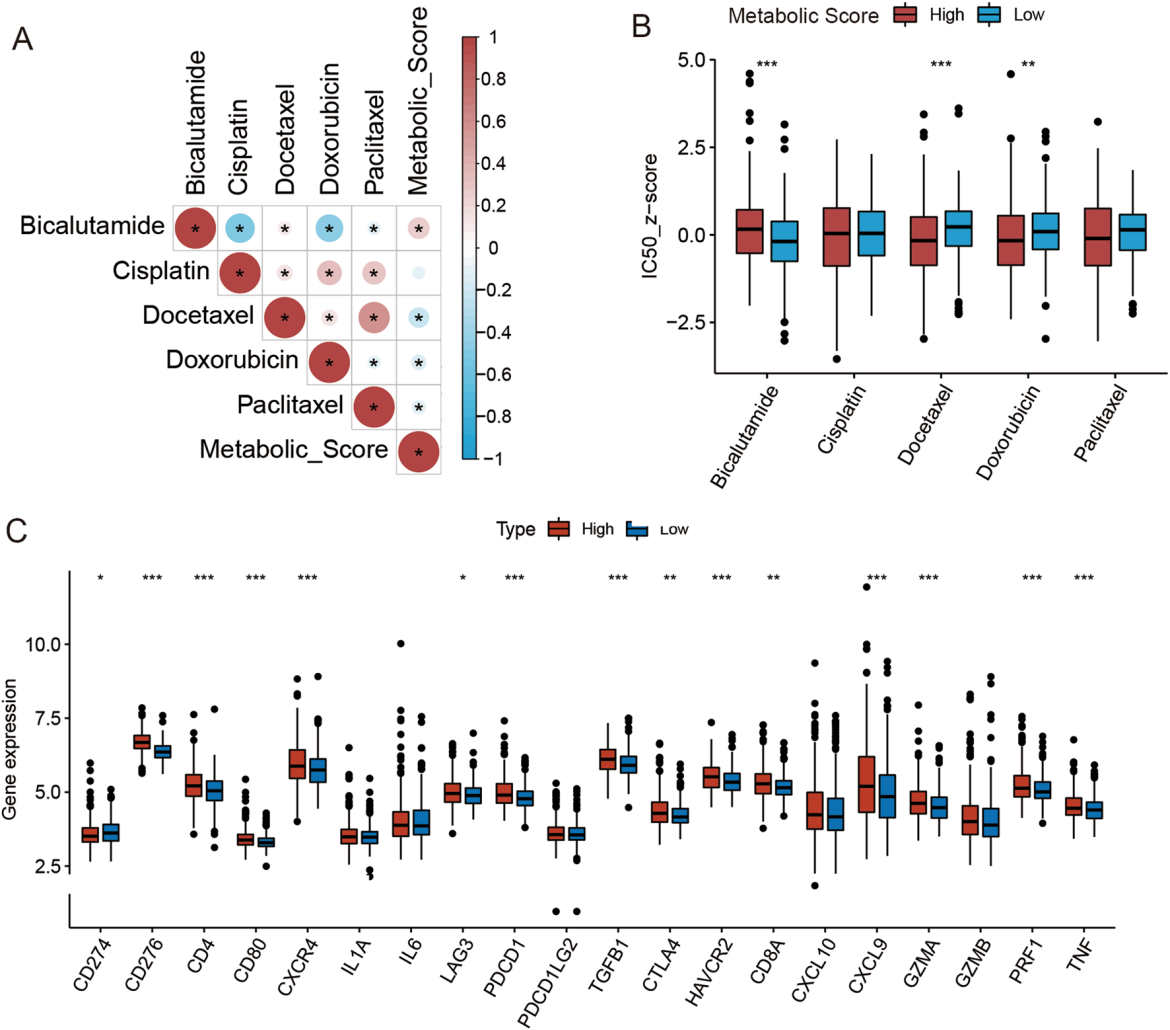
The drug-sensitive analysis. (**A**) The heatmap of the correlation between metabolic score and IC50 of ant-tumor drugs in TCGA cohort. (**B**) Boxplot of IC50 of ant-tumor drugs for low and high metabolic score groups in the TCGA cohort. (**C**) The differences of checkpoints in the high and low metabolism-score subgroups. Wilcoxon test. *p < 0.05; **p < 0.01; ***p < 0.001.

For metastatic prostate cancer and recurrent prostate cancer, androgen deprivation therapy is the main therapy and can significantly improve the prognosis^27^. Therefore, we analyzed the relationship between the metabolic score and drug sensitivity of bicalutamide in the TCGA cohorts. The results suggest that tumor samples in the high metabolic score group had a higher IC_50_ with bicalutamide than the samples in the low metabolic score group (Wilcoxon test: *P* < 0.001) (Fig. 7A and B). Docetaxel is the most common anti-tumor drug for castration-resistant prostate cancer (CRPC) and is, in general, the most common anti-tumor drug^28^. We executed the drug-sensitive analysis of docetaxel in TCGA cohorts and found that tumor samples in the high metabolic score group had a lower IC_50_ with Docetaxel than the sample in the low metabolic score group (Wilcoxon test: *P* < 0.001) (Fig. 7A and B).

Immune therapy is the most popular treatment for many cancers, and its effect is associated with the expression of checkpoint genes^29^. Although prostate cancer is an immune desert tumor, some studies display prospect of immunotherapy in PCa^30,31^. Therefore, we compared the expression levels of checkpoint genes between the two groups. The high metabolic score group had higher expression levels of *CD276, CD4, CD80, CXCR4, LAG3A, PDCD1, TGFB1, HAVCR2, CTLA4, CXCL9, CD8A, GZMA PRF1,* and *TNF* and lower expression levels of *CD274* compared to the low metabolic score group, indicating that patients in the high metabolic score group could benefit more from immunotherapy (Fig. 7C).

## Discussion

With the development of molecular biology and targeted therapy, particularly castration and androgen-deprivation therapy, the prognosis of PCa patients has improved remarkably^32^. However, because all recurrent PCa patients will develop CRPC after castration and antimale treatment, a new biomarker is needed to predict PCa prognosis and guide drug treatment^5^. Through ssGSEA, unsupervised clustering, PCA, univariate and multivariate cox analysis, we constructed a metabolic score and found that it was closely associated with DFS and reflected the therapeutic effectiveness of anti-tumor therapy. We validated our metabolic score in six datasets and more than 1000 samples of PCa to prove that our results are credible and valuable.

In a previous study, we found that metabolic change was a significant characteristic between normal prostate tissue and prostate cancer tissue^22,33^. To further explore the metabolic features of PCa samples, we performed ssGSEA of metabolic pathways and found that patients with PCa samples of low metabolism had the worst prognosis. Prostate is a smooth muscle tissue with an inherently high metabolism, and the function of prostate tissue as well as changes and the metabolic status of the sample will decline when tissue undergoes canceration^7,34,35^. In primary PCa, the PCa sample has a high metabolic level and the best prognosis because of a low heterogeneity. In advanced PCa with high heterogeneity, the metabolic levels in the samples with respect to a normal metabolism will markedly diminish, and some special metabolic pathways will increase to counter this phenomenon and continue providing energy to cancer cells^36^. Therefore, these patients had the worst prognosis. At the same time, we used metabolic biomarkers to predict PCa prognosis based on bioinformatics and tested the biomarkers in multiple cohorts.

Many studies have reported that DNA mutations are the driving factor of tissue canceration and decrease the prognosis of patients, including PCa^37,38^. At the same time, TMB is a recognized biomarker of anti-tumor immune therapy^29,39,40^. We found that the metabolic score had a close relationship with DNA mutation, CNV, and MSI. Based on the low metabolically active samples at an advanced stage, these samples had accumulated DNA mutations and may be more sensitive to immune therapy. Furthermore, we found that low and high metabolic score samples had different gene-related DNA mutations, indicating that the metabolism subtype of PCa is not only a phenomenon related to the accumulation of DNA mutations, but also the two developmental orientations of PCa. Therefore, the metabolic score predicted the value of patient prognosis in different stages of PCa. Meanwhile, it is possible to further explore the reason for why the different metabolic samples had different DNA mutation spectra to understand the mechanism of development and appearance of PCa. In addition, these genes may be potential drug targets for anti-tumor therapy.

With the progress of tumor immunology and the rise of immune therapy of tumors, TME and immune cells play a crucial role in tumors^41–43^. In some clinical trials, immune checkpoint block therapy has been reported successful for many cancers, featuring a significant curative effect^44,45^. In our study, we determined that the metabolic score could reflect tumor TME. High metabolic score tumors have a higher content of stromal cells and certain immune cells than low metabolic score tumors. We assume this indicates that some stromal cells were carcinogenic. For example, EMT^46^, which led to a significant increase in the high metabolic score group, was reported to promote cancer progression and transfer. At the same time, we believe that the increase in immune cells resulted from an increase in tumor heterogeneity, and some immune cells showed a decline in the high metabolic score group because of the depletion of the immune system. Based on the high immune infiltration, high expression of immune checkpoint genes, and high TMB, we believe a patient with a high metabolic score may benefit more from immune therapy more than other patients.

At present, castration and antimale therapy are the first-line treatments for metastatic prostate cancer^27^. As for CRPC, docetaxel is often used as an anti-tumor treatment^28^. In considering TMB, TME, and tumor immunity, we further analyzed the drug sensitivity associated with the metabolic score based on the Genomics of Drug Sensitivity in Cancer (GDSC). Through machine learning and forest regression analysis using the “pRRophetic” R package, we predicted the IC50 of each anti-tumor drug for each sample based on tumor mRNA expression data. We found that samples with a high metabolic score had higher IC50 of bicalutamide than those with a low metabolic score. At the same time, further analysis of the AR expression revealed that samples with high metabolic scores had higher AR expression levels than those with low metabolic scores. Hence, we assume that patients with high metabolic scores have low sensitivity to bicalutamide, possibly due to the high expression of AR, which would require a higher concentration of bicalutamide to antagonize ARs and inhibit the growth of PCa. For other chemotherapy drugs, low-metabolic-score samples were more insensitive because these samples could metabolize the drugs at rapid rates following anti-tumor therapy^47,48^. In summary, our metabolic score is associated with the drug sensitivity to anti-tumor therapy and can potentially direct clinicians toward choosing a suitable treatment for PCa.

Compared with other studies featuring public datasets, our work is mainly concerned with a larger number of datasets and samples. To avoid the instability of the bioinformatic analysis results, almost all analyses were performed in at least two independent cohorts. Furthermore, the metabolic score obtained by PCA reflected more biomarker features and could more accurately reflect tumor heterogeneity than traditional risk models built using cox analysis can. Finally, we used a multi-omics dataset to explore disease mechanisms. Therefore, we believe our results are stable and accurate. However, our study has some limitations. First, the reaction rate of the PCa anti-tumor therapy cohort was not accessible. The predictive value of the metabolic score for anti-tumor therapy in PCa needs further verification. Second, because of the batch effect, the metabolic score cannot be used to directly compare different cohorts without batch correction.

## Conclusion

We established a metabolic score based on a large number of samples, multiple databases, and sophisticated bioinformatics calculations to describe the metabolic characteristics of PCa tissue. Our metabolic score could predict patient prognosis and guide anti-tumor therapy in PCa. In addition, we discovered that DNA mutations might be the driving factor that leads to metabolic disorders in PCa. Finally, metabolic characteristics were found to be associated with TME and immune infiltration in PCa. Although our results need further validation by experimental confirmation, they provide valuable insights into the mechanism and clinical treatment of PCa.

## Supplementary Information

Supplementary Figure 1.

Supplementary Figure 2.

Supplementary Figure 3.

Supplementary Figure 4.

Supplementary Figure 5.

Supplementary Figure 6.

Supplementary Figure 7.

Supplementary Figure 8.

Supplementary Figure 9.

Supplementary Figure 10.

Supplementary Table 1.

Supplementary Table 2.

Supplementary Table 3.

Supplementary Table 4.

Supplementary Table 5.

Supplementary Table 6.

Supplementary Table 7.

Supplementary Table 8.

Supplementary Table 9.

Supplementary Legends.

## Data Availability

The data analyzed in this study can be downloaded from the TCGA, cBio, and GEO.

## Abbreviations

AR: Androgen receptor
CAPRA: Cancer of the prostate risk assessment
CNV: Copy number variation
CRPC: Castration-resistant PCa
DEGs: Differentially expressed genes
DFS: Disease free survival
DKFZ: Deutsches krebsforschungszentrum
ECM: Extracellular matrix
EMT: Epithelial-mesenchymal transition
FPKM: Fragments per kilobase per million
GEO: Gene expression omnibus
GO: Gene ontology
KEGG: Kyoto encyclopedia of genes and genomes
LogFC: Log fold change
MSigDB: Molecular signatures database
MSKCC: Memorial sloan-kettering cancer center
NCCN: National comprehensive cancer network
PCa: Prostate cancer
PCA: Principal component analysis
ssGSEA: Single-sample gene set enrichment analysis
TCGA: The cancer genome atlas
TGF-β: Transforming growth factor-beta
TMB: Tumor mutation burden
TPMs: Transcripts per kilobase million
