# Supplementary figures and images for: Metabolic characterization and metabolism-score of tumor to predict the prognosis in prostate cancer
Source: Sci Rep. 2021 Nov 18;11:22486. doi: 10.1038/s41598-021-01140-6 (PMC8602249; doi:10.1038/s41598-021-01140-6)

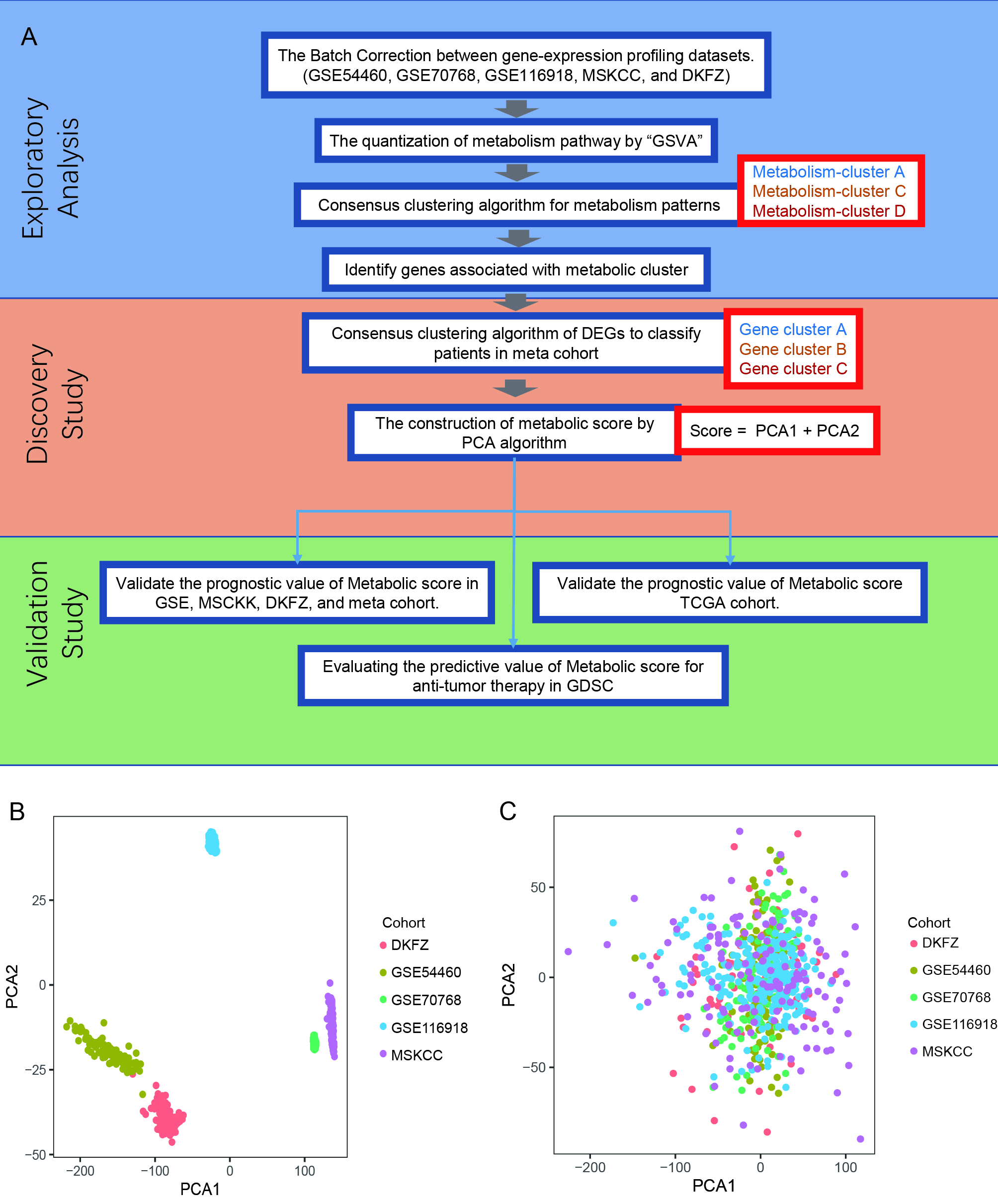

Supplement: Supplementary file 1 — Supplementary Figure 1. [file 41598_2021_1140_MOESM1_ESM.tif]

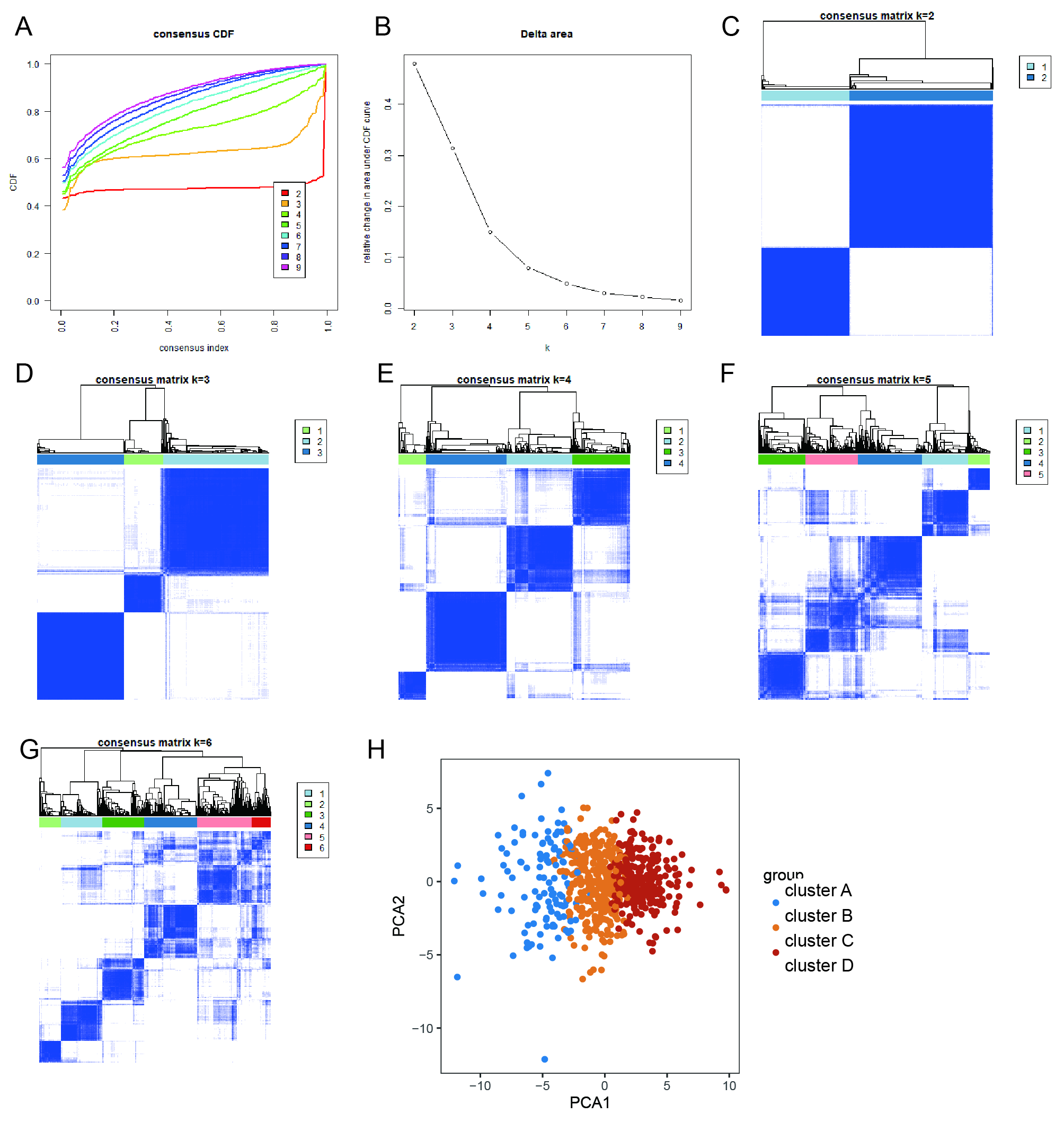

Supplement: Supplementary file 2 — Supplementary Figure 2. [file 41598_2021_1140_MOESM2_ESM.tif]

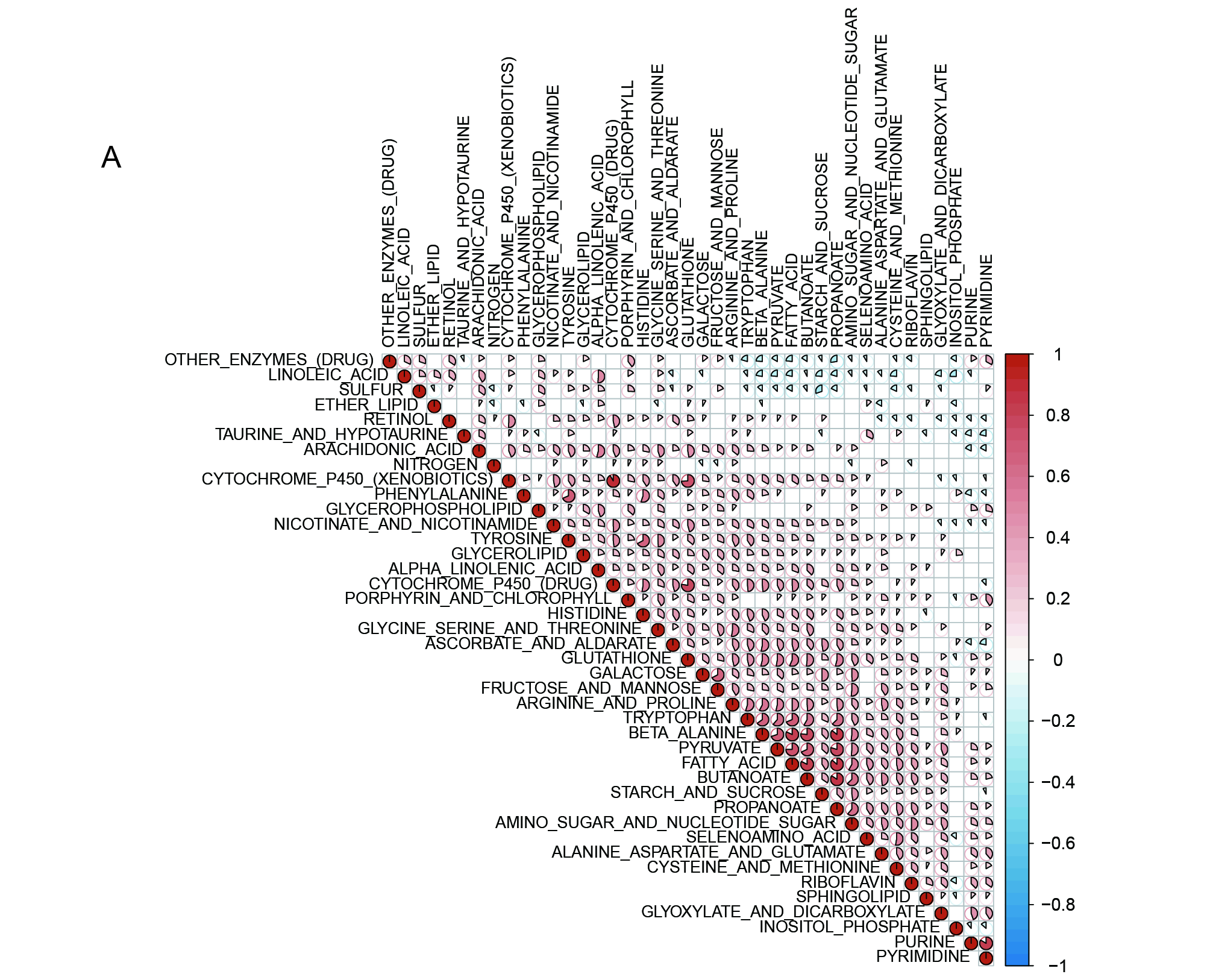

Supplement: Supplementary file 3 — Supplementary Figure 3. [file 41598_2021_1140_MOESM3_ESM.tif]

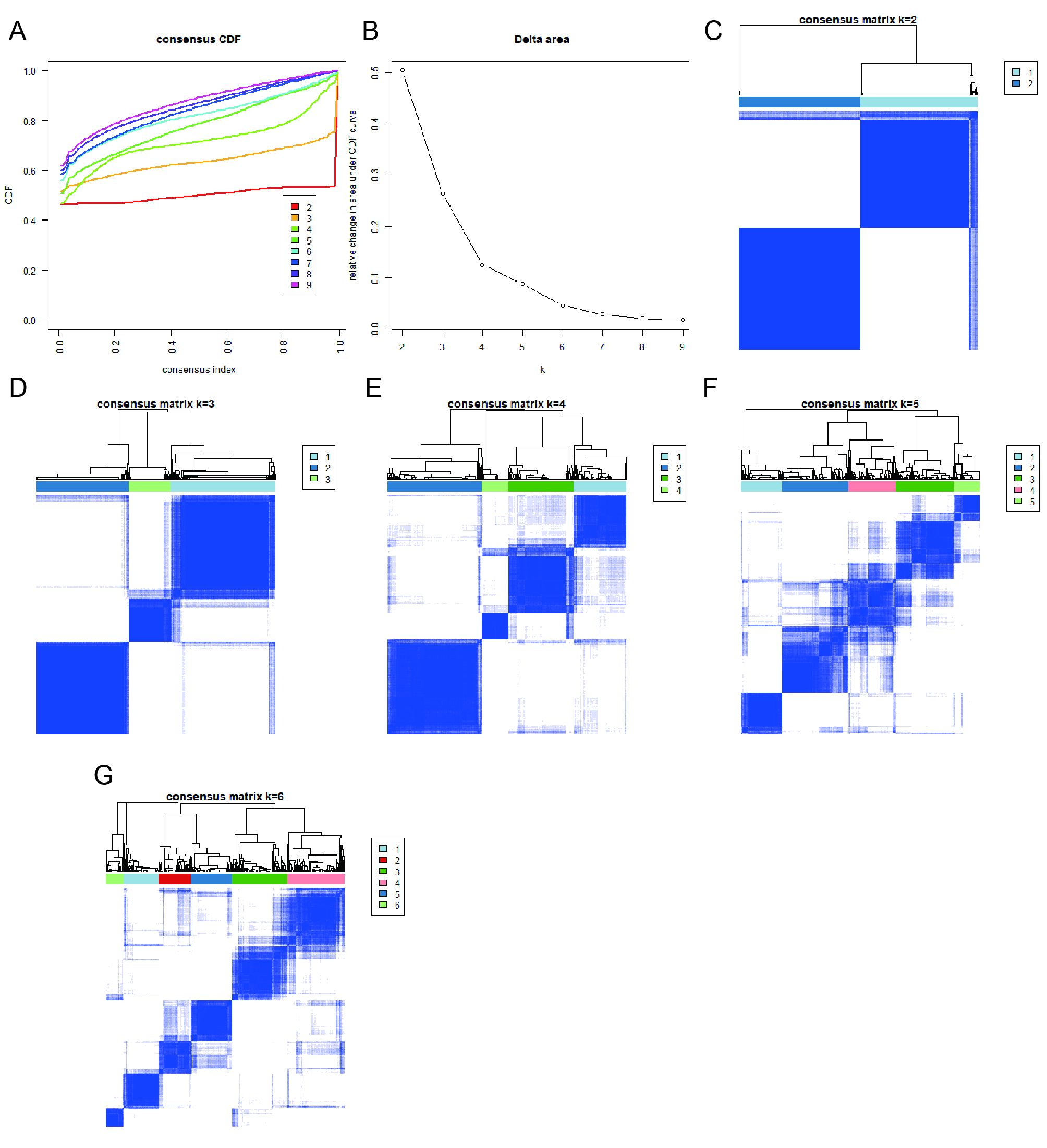

Supplement: Supplementary file 4 — Supplementary Figure 4. [file 41598_2021_1140_MOESM4_ESM.tif]

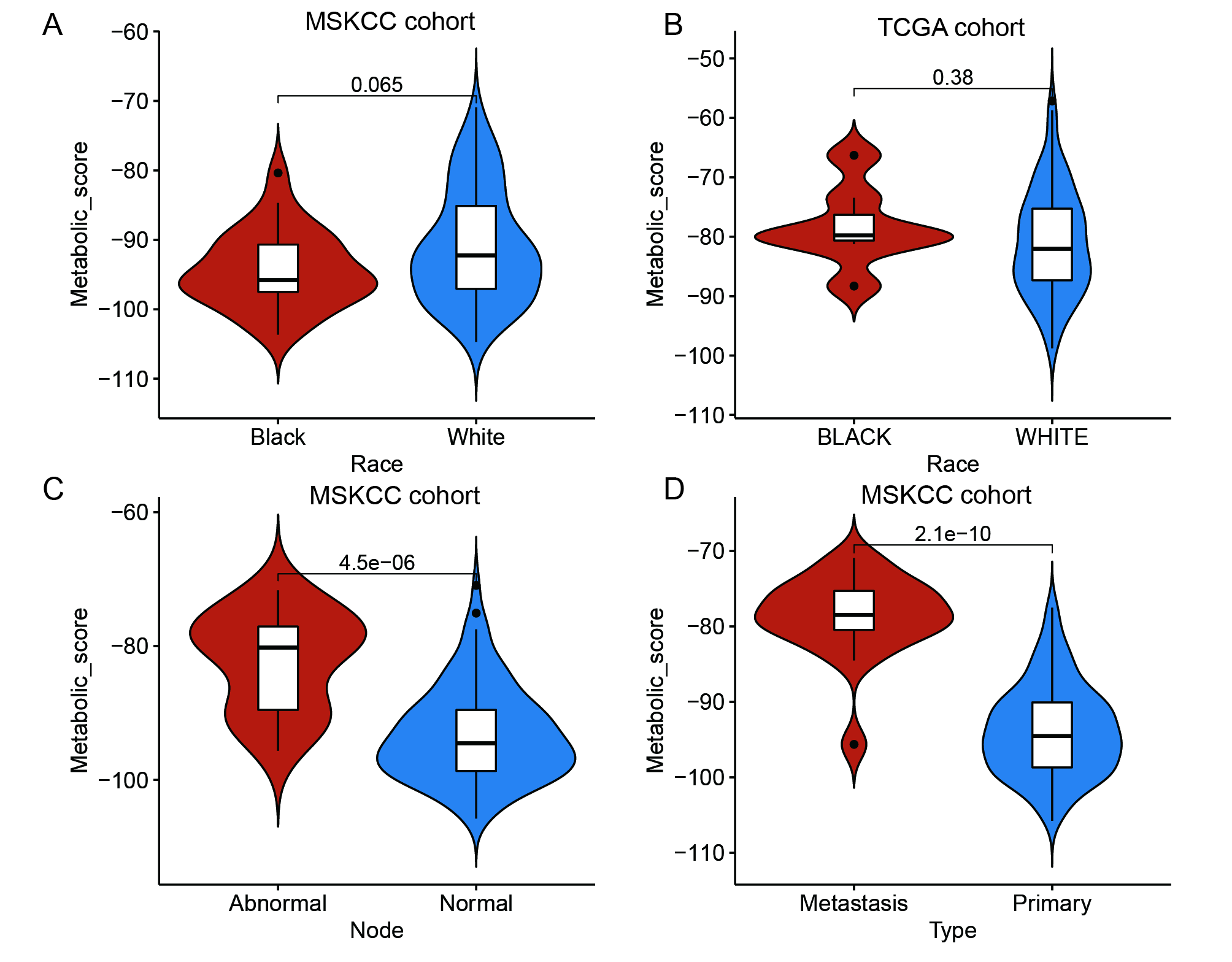

Supplement: Supplementary file 5 — Supplementary Figure 5. [file 41598_2021_1140_MOESM5_ESM.tif]

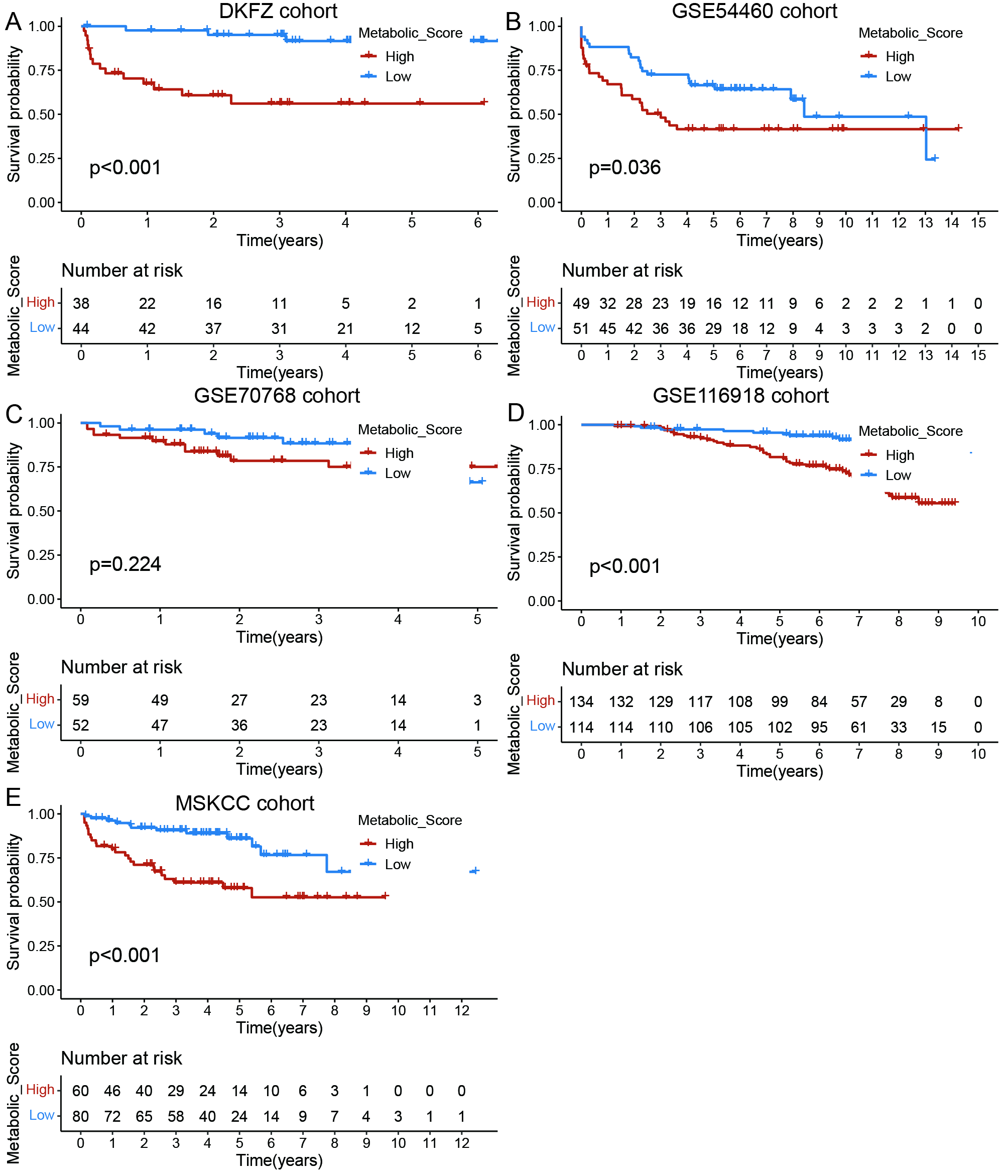

Supplement: Supplementary file 6 — Supplementary Figure 6. [file 41598_2021_1140_MOESM6_ESM.tif]

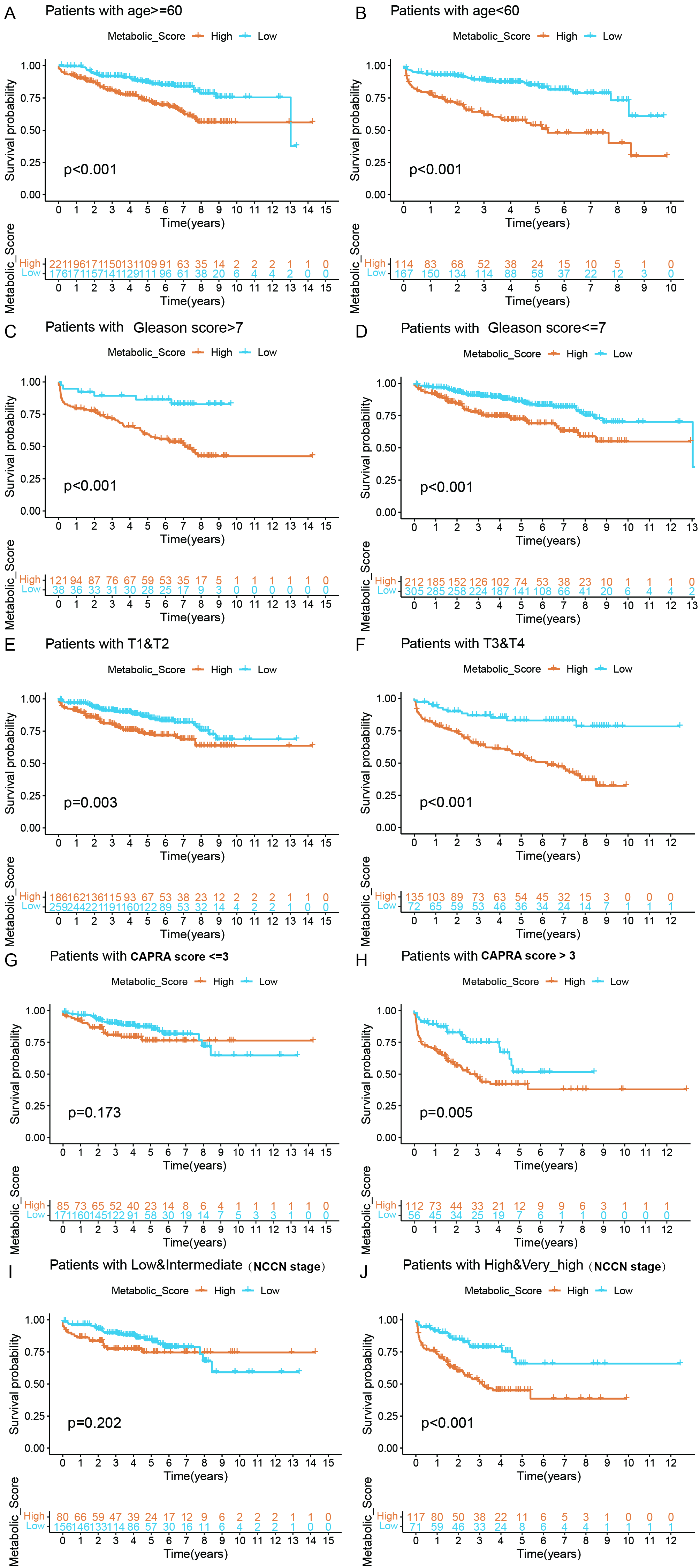

Supplement: Supplementary file 7 — Supplementary Figure 7. [file 41598_2021_1140_MOESM7_ESM.tif]

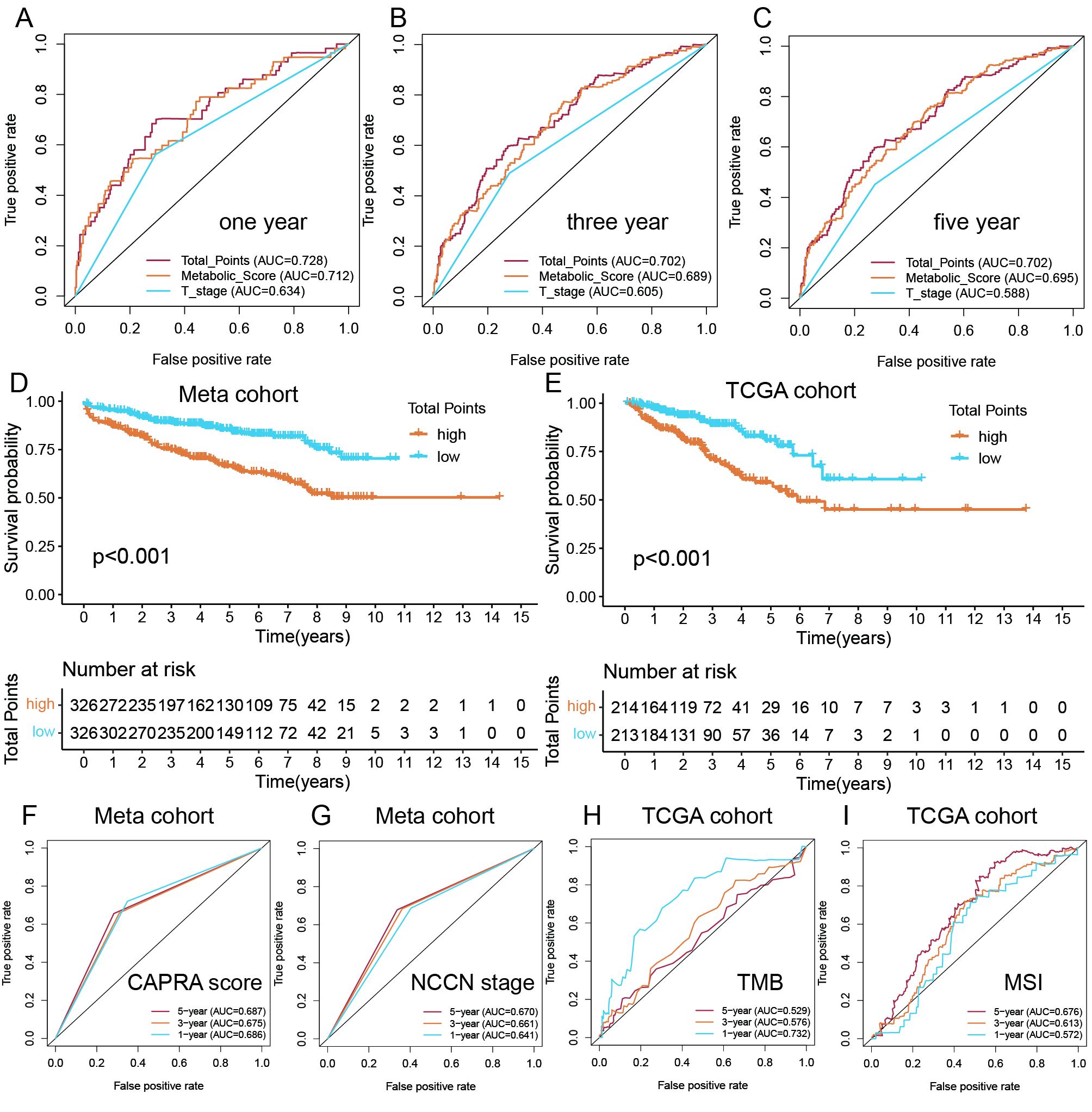

Supplement: Supplementary file 8 — Supplementary Figure 8. [file 41598_2021_1140_MOESM8_ESM.tif]

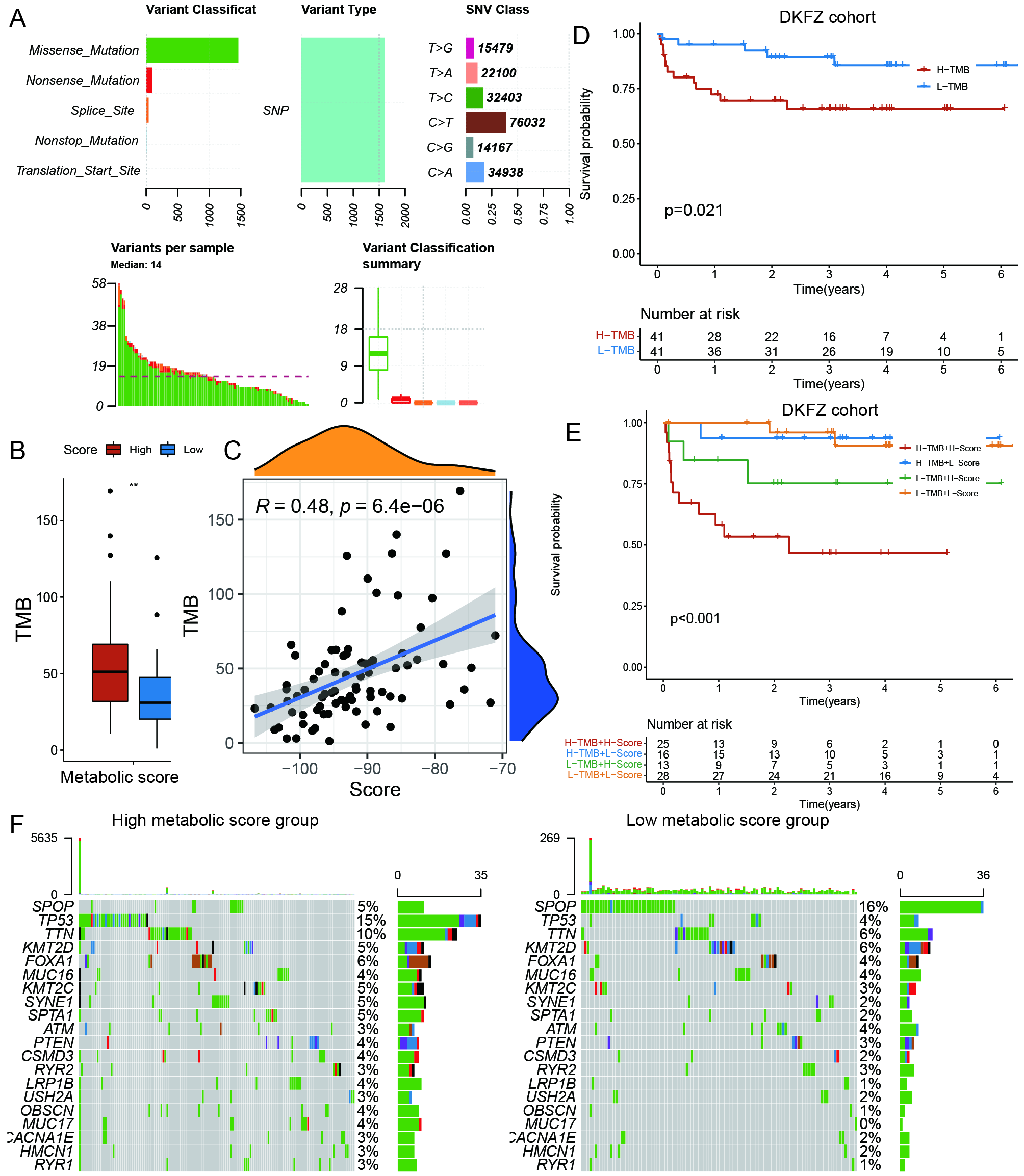

Supplement: Supplementary file 9 — Supplementary Figure 9. [file 41598_2021_1140_MOESM9_ESM.tif]

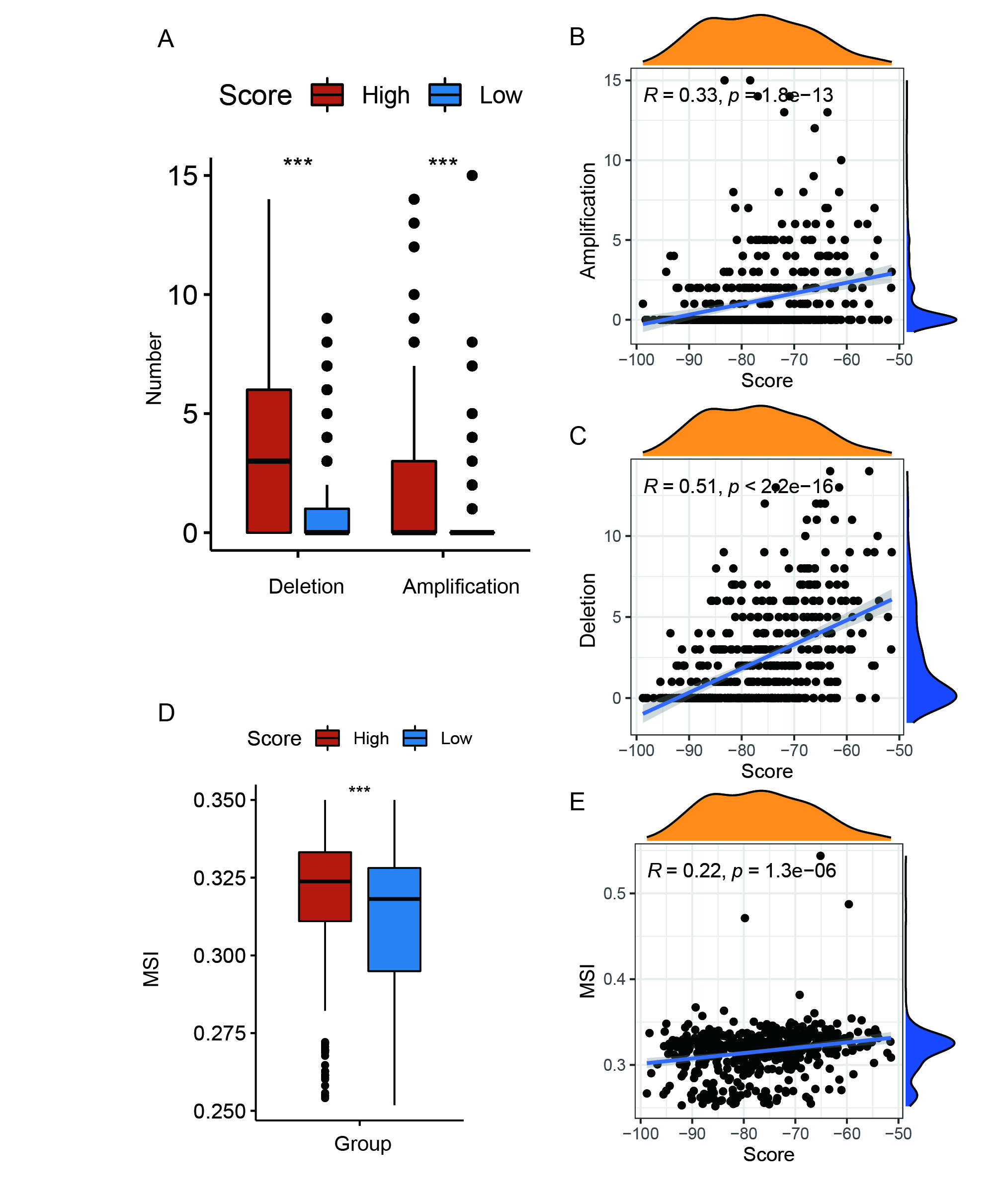

Supplement: Supplementary file 10 — Supplementary Figure 10. [file 41598_2021_1140_MOESM10_ESM.tif]
