# Supplementary material for: Metabolic characterization and metabolism-score of tumor to predict the prognosis in prostate cancer
Source: Sci Rep. 2021 Nov 18;11:22486. doi: 10.1038/s41598-021-01140-6 (PMC8602249; doi:10.1038/s41598-021-01140-6)
Supplement: Supplementary file 20 — Supplementary Legends. [file 41598_2021_1140_MOESM20_ESM.docx]

Supplemental Figure 1: (A) Overview of study design. (B) Principal Component Analysis (PCA) of mRNA expression data of sample from five cohorts before batch correction. (C) Principal Component Analysis (PCA) of mRNA expression data of sample from five cohorts before batch correction.

Supplemental Figure 2: (A-G) Consensus matrixes of PCa samples for each k (k = 2–6) from meta cohort based on metabolic ssGSEA score, displaying the clustering stability using 1000 iterations of hierarchical clustering. (H) Principal Component Analysis (PCA) of meta cohort based on metabolic ssGSEA score.

Supplemental Figure 3: The correlation of the metabolic ssGSEA score of PCa samples.

Supplemental Figure 4: (A-G) Consensus matrixes of PCa samples for each k (k = 2–6) from meta cohort based on DEGs expression abundance, displaying the clustering stability using 1000 iterations of hierarchical clustering.

Supplemental Figure 5: The differences of metabolic score between black and white races in MSKCC cohort (A) (Wilcoxon test, p = 0.065) and TCGA cohort (B) (Wilcoxon test, p = 0.038), and between different Node stages (C) (Wilcoxon test, p < 0.001) and metastasis stages (D) (Wilcoxon test, p < 0.001) in MSKCC cohort.

Supplemental Figure 6. Kaplan-Meier curves for the low/high metabolic score groups in DKFZ cohort (A) (Log-rank test, p < 0.001), GSE54460 cohort (B) (Log-rank test, p = 0.036), GSE70768 (C) (Log-rank test, p = 0.224), GSE116918 (D) (Log-rank test, p < 0.001), and MSKCC (E) (Log-rank test, p < 0.001).

Supplemental Figure 7. Hazard stratification analysis in meta cohort. age >= 60 (A) ((Log-rank test, p < 0.001), age < 60 (B) (Log-rank test, p < 0.001), Gleason score > 7 (C) (Log-rank test, p < 0.001), Gleason score <= 7 (D) (Log-rank test, p < 0.001), T1-T2 (E) (Log-rank test, p < 0.001), T3-T4 (F) (Log-rank test, p < 0.001), CAPRA score <= 3 (G) (Log-rank test, p < 0.001), CAPRA score > 3 (H) (Log-rank test, p < 0.001), low & intermediate NCCN stages (I) (Log-rank test, p < 0.001), and high & very high NCCN stage (J) (Log-rank test, p < 0.001).

Supplemental Figure 8. The predict value of nomogram. The ROC analysis for nomogram in 1-year (A), 3-year (B), and 5-year (C). Kaplan-Meier curves for the low/high total points groups in meta (training) (D) cohort and TCGA (validation) (E) cohort. The ROC analysis for CAPRA score (F), NCCN stage (G), TMB (H), and MSI (I) in 1-year, 3-year, and 5-year.

Supplemental Figure 9: The Correlation between the metabolism-score and somatic variants in DKFZ cohort. (A) The summary information of Somatic Variants. (B) TMB difference in the high and low metabolism-score subgroups. Wilcoxon test, p<0.001. (C) Scatterplots depicting the negative correlation between metabolism-scores and mutation load. The Spearman correlation between metabolism-scores and mutation load is shown (p<0.001). (D) Kaplan-Meier curves for high and low TMB groups. Log-rank test, p=0.021. (E) Kaplan-Meier curves for patients stratified by both TMB and metabolism-scores. Log-rank test, p=0.020. (F) The oncoPrint was constructed using high metabolism-scores on the left and low metabolism-scores on the right. Individual patients are represented in each column.

Supplemental Figure 10: (A-C) The Correlation between the metabolism-score and CNV in TCGA cohort. (D-E) The correlation between the metabolism-score and MSI in the TCGA cohort.

Supplemental Table 1: Clinical information of PCa patients from six cohorts.

Supplemental Table 2: Gene sets analyzed in the article.

Supplemental Table 3: The univariate cox analysis and K-M survival analysis of ssGSEA score of 41 metabolic pathways in meta cohort.

Supplemental Table 4: DEGs between metabolism clusters

Supplemental Table 5: Univariate cox analysis of prognostic DEGs.

Supplemental Table 6: GO and KEGG analysis of DEGs gene cluster related genes

Supplemental Table 7: PCA analysis and PCA coefficient to calculated metabolic score.

Supplemental Table 8: GSEA analysis between high and low metabolism-score group.

Supplemental Table 9: Univariate and multivariate cox analysis of clinical variate and metabolic score.
